# Sexual abstinence: What is the understanding and views of secondary school learners in a semi-rural area of North West Province, South Africa?

**DOI:** 10.1080/17290376.2016.1195281

**Published:** 2016-06-17

**Authors:** Kebogile Mokwena, Mamaponesa Morabe

**Affiliations:** ^a^ EdD, Department of Public Health, Sefako Makgatho Health Sciences University, Pretoria, South Africa; ^b^ MPH, Department of Public Health, Sefako Makgatho Health Sciences University, Pretoria, South Africa

**Keywords:** HIV prevention, sexual abstinence, adolescents, barriers, focus groups, prévention du VIH, abstinence sexuelle, adolescents, obstacles, groupes de discussion

## Abstract

Among strategies to prevent HIV, other sexually transmitted infections (STIs) and unwanted pregnancies, are programs that promote sexual abstinence among adolescents. However, literature suggests that there may be differences in the understanding of abstinence across adolescents, and this study sought to explore the understanding of sexual abstinence among both male and female learners in a secondary school in a semi-rural area of North West Province, South Africa. Focus group discussions were used to collect data from learners who were in grades 8–10 at the time of the study. The findings are that the learners in this area understand sexual abstinence as the decision not to have sex, and this was associated with prevention of HIV, STIs and unwanted pregnancies, which ensures a better future. Barriers to sexual abstinence include peer pressure, myths and wrong perceptions about sex, influence of drugs and alcohol and the influence of television. Based on how it is delivered, school-based sex education was viewed as both an enabler and barrier to sexual abstinence. It is recommended that programs to promote sexual abstinence be strengthened and such programs be community-based.

## Résumé

Parmi les stratégies concernant la prévention du VIH, d’autres maladies sexuellement transmissibles (MST) et des grossesses non planifiées, il existe des programmes qui encouragent l’abstinence sexuelle parmi les adolescents. Toutefois, de nombreux documents suggèrent qu’il y aurait des différences en ce qui concerne la compréhension de l’abstinence parmi les adolescents, et cette étude a cherché à explorer la compréhension de l’abstinence sexuelle parmi les écoliers et les écolières d’une école secondaire dans une zone semi-rurale de la Province du Nord-Ouest, en Afrique du Sud. Des groupes de discussion furent utilisés pour récolter des données des écoliers qui étaient en quatrième, troisième et seconde pendant cette étude. Les résultats ont montré que les écoliers dans cette zone interprétaient l’abstinence sexuelle comme une décision de ne pas avoir de rapport sexuel, et ceci était associé à la prévention du VIH, STD et des grossesses non planifiées, qui garantissent un meilleur futur. Les obstacles à l’abstinence sexuelle incluent la pression des pairs, les mythes et les perceptions erronées concernant le sexe, l’influence des drogues et de l’alcool et l’influence de la télévision. Selon sa présentation, l’éducation sexuelle enseignée à l’école était considérée comme un facilitateur et un obstacle à l’abstinence sexuelle. Il est donc recommandé de renforcer les programmes promouvant l’abstinence sexuelle et de faire en sorte qu’ils incluent la communauté.

## Introduction

The past decade has seen an increase in the promotion of sexual abstinence programs, as efforts to curb the spread of HIV, other sexually transmitted infections (STIs) and unwanted pregnancies among adolescents (Kantor, Santelli, Teitler & Balmer 2008). The promotion of sexual abstinence enjoyed a boost at a time when initiation of sexual activity among adolescents has emerged as a major risk factor for negative reproductive health outcomes, including early childbearing and associated implications for maternal and child health outcomes, as well as increased risk for STIs, including HIV (Morrison-Beedy, Carey, Côté-Arsenault, Seibold-Simpson & Robinson 2008). Sexual abstinence is therefore often regarded as the opportune primary response to prevention of adolescent pregnancy and STIs (Morrison-Beedy *et al.* 2008; Ott, Millstein & Halpern-Felsher 2006).

One of the major success stories in changes in risk of sexual behavior and the resultant reduction of incidence of HIV in the African continent was Uganda, and their success has not been credited to use of condoms, but sexual abstinence (Barnett & Parkhurst 2005; Green 2003; Green, Halperin, Nantulya & Hogle 2006; Low-Beer & Stoneburner 2003; Stoneburner & Low-Beer 2004). This view is aligned with practices in many African communities, where tradition promotes the avoidance of premarital sex and childbearing by young people (Kabiru & Ezeh 2007). As a way of encouraging and enforcing sexual abstinence, some South African communities practice virginity testing, to promote sexual abstinence among female adolescents, to prevent unwanted pregnancies and the spread of HIV/AIDS (George 2008; Taylor, Dlamini, Sathiparsad, Jinabhai & de Vries 2007; Vincent 2006).

There is a lack of consensus on the success of abstinence-promoting programs in reducing risky sexual behaviors among young people, with some promoting it (Jemmott, Jemmott & Fong 2010) and others concluding that they are not effective in reducing the risk of HIV and that they are actually disadvantageous because they often undermine more comprehensive sexuality education by withholding some information on sexual health (Santelli, Ott, Lyon, Rogers, Summers & Schleifer 2006). However, the promotion of sexual abstinence remains a consideration and an option across the world, due to differences in culture, personal choices and religious convictions. Moreover, many of sexually experienced young people wish they had waited longer before having sex (Collins, Elliott, Berry, Kanouse, Kunkel, Hunter, *et al.* 2004).

The decision to practice sexual abstinence is shaped by a number of interlinking forces that include individual, family and community influences. Such influences may include what youth discuss and consider to be reality among themselves, and such reality may be different from that of other groups. Studies have also stated that definitions of ‘being abstinent’ may differ between rural or urban adolescents (Morrison-Beedy *et al.* 2008) and that other factors that determine understanding of abstinence include gender, age, ethnicity and sexual experience (Bersamin, Fisher, Walker, Hill & Grube 2007). Hans and Kimberly (2011) also concluded that conceptualizations of abstinence may be changing among young adults.

Abstainers may be classified as primary, secondary or recent. Primary abstainers are sexually inexperienced and have chosen not to have sex at all. For this group, ‘abstinence’ and ‘virginity’ can be used interchangeably. Secondary abstainers have some sexual experience, but have abstained for the past 12 months or more. These are often driven by a desire to avoid STIs, HIV, or pregnancy. Recent abstainers were sexually active in the last year but not in the past three months and may be abstaining due to lack of opportunity to engage in sexual intercourse rather than as a conscious choice to abstain (Kabiru & Ezeh 2007; Ott *et al.* 2006). The exposure to HIV and other STIs varies across these groups of abstainers.

The understanding of abstinence, its role and practice varies across cultures, communities and geographical regions (Bersamin, Walker, Waiters, Fisher & Grube 2005, 2007; Kabiru & Ezeh 2007). As an example, in South Africa, the social construction of young people’s sexuality is shaped, among others, by the contemporary resurgence of ‘traditionalism’ which is promoted across many communities (Harrison 2008). These variations are potential influences to the understanding, the practice and value of sexual abstinence. Although there is consensus on the importance of sexual abstinence in the prevention of STIs and pregnancy (Kabiru & Ezeh 2007), most abstinence programs are based on adult ideas of abstinence, and little is known about how adolescents themselves conceptualize sexual abstinence. It is for these reasons that the understanding of sexual abstinence among adolescents needs to be explored (Hans & Kimberly 2011).

Not only is the understanding of abstinence gender based (Lillie, Pulerwitz & Curbow 2009) but literature has shown that there are gender-based differences in the primary reasons for sexual abstinence. While female adolescents may be more motivated to abstain due to a desire to avoid pregnancy, male adolescents abstain because they avoid STIs/HIV (Kabiru & Ezeh 2007). The acceptance of abstinence as a prevention method for pregnancy and STIs for adolescents depends on their understanding of the concept, and since there are differences in young people’s understanding of abstinence (Bersamin *et al.* 2007; Dailard 2003), there is a need to explore this understanding among young people, hence this study.

## Objective

The objective of this study was to explore the understanding of sexual abstinence by learners attending a secondary school in a semi-rural area of North West Province.

## Study design

An exploratory, qualitative and descriptive design, using focus groups discussions, was used with a sample of secondary school learners, to explore their understanding and role of sexual abstinence in sexual behavior.

## Study setting, population and sample

The study population consisted of male and female learners, who attended a secondary school (grades 8–10) in Bojanala District, North West Province. This area was selected because it has a combination of learners from the urban township and semi-rural, and the data would give a combination of both groups of learners. Within this population, purposive sampling of learners aged 15 years and above, who were willing to participate in the study and whose parents provided informed consent, were included. The sample size was determined by data saturation, which is the stage where additional focus group discussions no longer provided new information. Data saturation was reached after conducting six focus group discussions.

## Recruitment

Participants were recruited from school during a life orientation class, which is compulsory for all learners. During the class, the researcher was given time to give a brief explanation of the study, and learners who were interested in participating were given information letters and informed consent forms that request their parents to give permission for the learners to participate in the study. Although all learners were recruited and had an equal opportunity to participate in the study, only learners who returned the signed informed consent forms were eligible to participate. Each focus group discussion consisted of participants who were in the same grade.

## Data collection

Data were collected using a researcher-developed focus group discussion guide, developed in English and translated into the local Setswana language. The focus group discussion guide was developed using a review of literature on the understanding and use of abstinence by adolescents. The focus group guide was pilot-tested at a high school in Ga-Rankuwa, a township whose learners are similar in terms of age group and language with the learners for the main study.

Data collection occurred in a classroom after the school program was completed, and this provided a quiet atmosphere with no distractions from other learners. Participation was limited to learners whose parents had provided the informed consent. Each focus group had both male and female learners numbering between six and eight. The purpose of the study was explained and the assent forms were signed by all participants. Demographic data were collected using a quantitative questionnaire. The researcher facilitated the discussions in English, but the participants were encouraged to respond in English, Setswana or a combination of both languages. All could use either language because English is the medium of instruction at school while Setswana is their mother tongue. A digital recorder was used to record the discussions. The duration of the focus group discussions ranged from one to one and half hours.

## Data analysis

The demographic data were descriptively analyzed. The qualitative data from the audio recordings were transcribed verbatim, translated from Setswana into English, typed into MS Word^®^ and uploaded into NVIVO 9 for analysis. A codebook with corresponding definitions was developed from the transcripts and these codes were applied to all the transcripts using Nvivo 9 software to identify and develop several themes. The themes formed the basis of the narrative.

## Trustworthiness

Trustworthiness was achieved by data triangulation, and by enhancing dependability of the findings. Data triangulation was implemented by collecting data from both male and female learners. Data triangulation was also achieved by the use of two independent coders (the student and the supervisor), discussing the codes and agreeing on the final codes. Dependability of the findings was enhanced by employing a digital recorder, transcribing the recorded data verbatim, prolonged engagement with the data, and by using Nvivo for data analysis.

## Ethical considerations

The proposal obtained ethics clearance from the Medunsa Research Ethics Committee (MCREC/H/167/2010:PG). Permission to conduct the study was obtained from the Education District Office, as well as from the management of the school. Informed consent was obtained from all parents whose children participated in the study, and assent was obtained from all participants.

## Demographics of the sample

The sample consisted of 50 participants, with 48% (*n* = 24) male and 52% (*n* = 26) female, who participated in six focus group discussions. Their ages ranged between 15 and 18 years, with a mean age of 17 years. The majority, 68% (*n* = 34) were sexually abstinent and 32% (*n* = 16) sexually active. The biggest group were in grade 10 at 46% (*n* = 23), followed by grade 9s at 38% (*n* = 19) and grade 8s at 16% (*n* = 8).

**Fig. 1.**
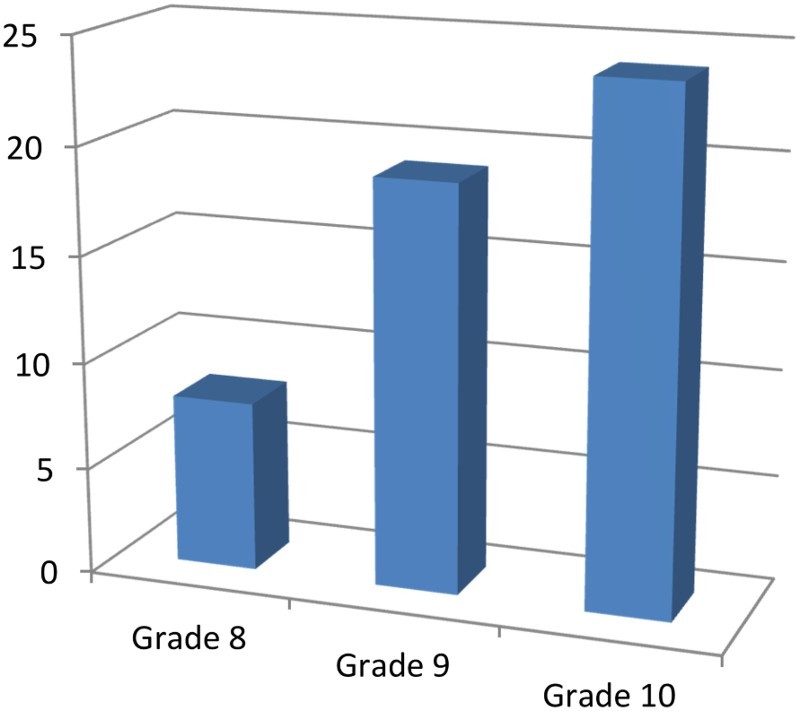
Grades of the participants.

**Fig. 2.**
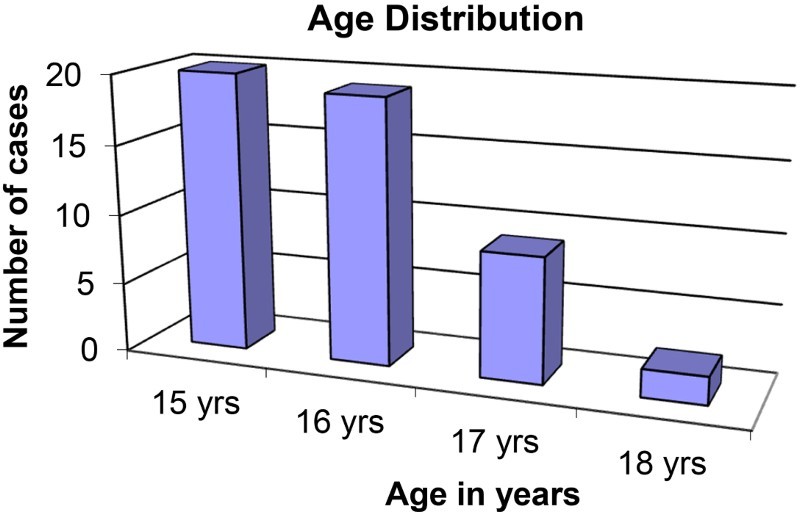
Ages of the participants.

**Fig. 3.**
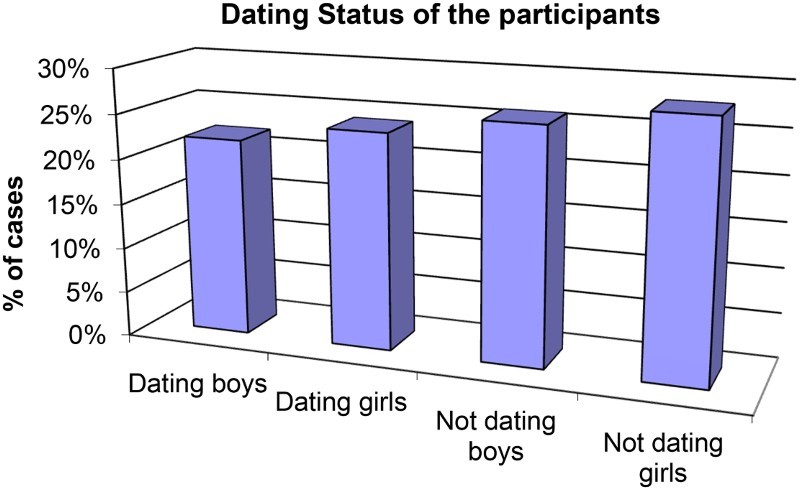
Dating status of the participants.

Figs. 1–3 show other demographic elements of the participants.

Dating was defined as being in a relationship with an individual of the opposite sex, and did not depict whether the participant was sexually abstinent or not. The percentages of the learners who were dating (46%) and the ones that were not dating at the time (54%) did not differ much. Of those who were dating, 48% were males and 52% were females.

## Qualitative findings

Eight themes emerged from analysis of the data, these being *understanding of sexual abstinence*, *abstinence as protection from sexually infectious diseases and unwanted pregnancies*, *abstinence ensures a better future*, *barriers to practicing abstinence*, *school-based sex education working against promotion of abstinence*, *emotional protection as an enabler to being abstinent*, *promoters of abstinence* and *the community as a resource to promote abstinence*.

### Theme 1: Understanding of sexual abstinence

This theme refers to what participants understand by abstinence. There is adequate consensus on what it is and the researcher did not attempt to differentiate between primary, secondary or recent abstinence. Asked about what abstinence means, the participants did not define or explain it, but went on to state the use, role and advantages of abstaining from sex. However, phrases like ‘abstain from sex’, ‘stop sex’ and ‘don’t want to have sex’, indicated that they understood abstinence as ‘not having sex’.

### Theme 2: Abstinence as protection from sexually infectious diseases and unwanted pregnancies

This theme refers to the understanding that abstinence is a way of protecting one from sexually transmitted diseases, including HIV, and unwanted pregnancies. This view, which was communicated by both males and females, seems to be the most frequently stated in relation to the use and value of sexual abstinence. Statements to substantiate this view included ‘Sexual abstinence is to protect yourself in life so that you won’t have problems such as contracting lots of diseases … ’ (male participant), ‘I think that teenagers abstain from sex and try to protect themselves because when you have sex you have to know that there are consequences, it’s either you get infected with STDs or you get pregnant … ’ (female participant).

Abstinence as a specific protection against HIV were also stated as follows: ‘ … we can use abstinence as a preventative measure because AIDS is everywhere, and mostly people get infected via engagement in sexual activities, and sex also causes teens to have unwanted pregnancies … ’ (male participant) and ‘abstinence is a preventative measure because teenagers would not have sex and they won’t be infected with HIV … ’ (male participant).

Within the discussion that abstinence provides protection from sexually infectious diseases and unwanted pregnancies, there was also a view that abstinence is better than other contraception methods, which was stated by both males and females as follows: ‘ … and contraceptives have their own consequences, so mostly contraceptives are not good, so sexual abstinence is the best measure … ’ (female), and a male stated it as follows: ‘Yes, I think abstinence is right as a preventative measure because mostly when girls talk about prevention, they automatically imagine contraceptives. Sexual abstinence will decrease teen pregnancies and many diseases … ’.

### Theme 3: Abstinence ensures a better future

This theme associates abstinence with a better future, which may be frustrated by the negative consequences of being sexually active and the resultant STIs and/or unwanted pregnancy. A better future was viewed as successful schooling and securing a career. The negative consequences of being sexually active, specifically in relation to acquiring STIs and unwanted pregnancies, were seen as threats to a bright future, and abstinence was therefore viewed as one thing that will secure or enable the desired bright future. The following statements are examples of how this view was stated: ‘ … But what is more important is that you can prevent many problems and be able to protect your future … ’ (male participant), ‘ … teenagers don’t want to contract diseases and they want to reach their future goals … ’ (female), ‘ … Some want to reach their goals and they think if they can have sex now and get pregnant they won’t be able to reach their goals … ’ (female participant) and ‘when you abstain from sex it means that you have a dream that you want to achieve … ’ (male participant).

### Theme 4: Barriers to practicing abstinence

This theme refers to people or situations that make it difficult for young people to abstain from sex, despite the acknowledged advantages of abstinence. Four sub-themes emerged from this theme, these being *peer pressure*, *myths and perceptions about sex*, *use of alcohol and drugs* and *influence of the media and television*.

#### Peer pressure

This sub-theme refers to the view that friends influence and exert pressurize among each other to engage in sexual activities, as illustrated by the following: ‘ … Teenagers usually get peer-pressure from their friends. They have sex because their friends have sex’ (male participant), and

I also say it is friends because we talk as friends about what we do in our relationships with our partners. So we judge each other with how often we have sex and that makes us to end up impregnating a girl … (Male participant)

#### Myths and perceptions

This sub-theme refers to myths that are often repeated among young people, which promote engagement in sexual activities.

‘ … they tell you that if you don’t engage in sexual activities you’re a stupid and you won’t get enough experience with sex and even when you’re married your partner will divorce you because of lack of experience in sex … ’ (female participant), ‘ … your friends will come to you and tell you to go have sex just like they did and that after sex you will look more beautiful … ’ (male participant) and

… because they tell you that if you are a virgin now then you’ll remain as you are and when you reach the age of 18 it will be impossible for you to have sex because your vagina will be blocked … (Female participant)

There are also attempts to ridicule virgins and uplift those that are sexually active or even promiscuous.

I spend most of my time with boys that like to hurt each other’s feelings by saying if you are a virgin you are stupid, so those who have sex with lots of girls they call themselves ‘Generals’ and us who don’t have sex they call us ‘Idiots’. Sometimes you wish to be like them and you want to try having sex too … (Male participant)

#### The influence of alcohol and drugs

Theme sub-theme refers to the view that the use of alcohol and drugs poses a challenge and often results in young people engaging in sex even though they would want to be abstinent. This view was stated as follows:

‘ … I think is when you went to a party and end up drinking alcohol, after you are drunk you lose control of yourself … ’ (female), ‘I think it is the taverns, when girls are drunk they end up leaving with people they don’t know. When that person buys you alcohol, you must repay him with your body, by having sex with him … ’ (female) and ‘Lots of boys come to school after smoking “nyaope” and they make you smoke it too and you end-up having sex with them … ’ (Female)

#### The influence of the media and television

This sub-theme identified the sexual content of the media and television as enablers to being sexually active.

Their views were stated as ‘Mostly, teenagers see sex on TV and they also want to have sex just as they saw it on TV … ’ (female participant). A male participant stated it as follows: ‘ … teenagers start sending each other pornographic videos with their cell phones and end-up wanting to do what they see on the videos, and have sex … ’

### Theme 5: School-based sex education working against promotion of abstinence

School-based sex education refers to the content of sex education that is part of the school curriculum, and is taught at school, mostly in a formal life orientation period. Some participants were of the view that conversations and information about sex, including at school, raise their curiosity and intentions to experiment with sex. The following statements illustrate this:

… Like at school we are being taught about sex, so you end-up wanting to experience about this thing they teach you about so much, it’s like that … (Male participant)

Yes at school they never said we must go and have sex but they teach us about it, therefore teenagers want to know why they are being taught about sex every day, because even if I don’t want to do it now I end-up gaining interest to know how it feels. So I want to know if what they teach me it’s true or not … (Male participant)

### Theme 6: Emotional protection as an enabler to being abstinent

This theme refers to both the risk of potential emotional harm that might result from being in a sexual relationship, and the emotional turmoil or guilt that comes with being sexually active. The participants showed the knowledge and understanding of the consequences of being sexually active. This is illustrated by the following statements:

Things that make teenagers to say they don’t want to have sex mostly is that, if you have been hurt by the first partner and the second partner also hurt you it’s then you realise that they didn’t love you and you start to pull back from sexual activities … (Female participant)

… because the relationship is not progressing or you see that you might get hurt or because of many diseases, or because your partner wants sex and you don’t … (Female participant)

### Theme 7: Promoters of abstinence

This theme refers to who the participants view as promoters of abstinence, and these included parents, teachers, guardians and the Christian religion, as reflected by the following quotes:

Sometimes teens listen to their mothers when the mother says ‘don’t do this’ they don’t do it because they know that there are consequences … (Female participant)

Also at the schools and clinics and our parents tell us too, especially if your parent is a nurse like myself, she will tell me that she sees people who have AIDS daily and that after AIDS you can’t survive … (Female participant)

… Even at home they tell me always that if I fall pregnant I am on my own they are not going to help me. So I have to keep that in my mind that I have to engage in sexual activities when I finish school … (Female participant)

Many things that make people to stay abstinent is teachers, especially the Life Orientation teachers who teach children about sex and also that sex is not good for children … (Male participant)

Sexual abstinence helps because when you read the bible it says ‘sex before marriage is a sin’ so if you are afraid of God you will know things that you don’t have to do … (Female participant)

Sometimes going to church is right because during the youth ministries you get informed and they tell us that we are not supposed to have sex before marriage and we also help each other with opinions … (Female participant)

### Theme 8: Community as resource for the promotion of abstinence

This theme refers to the view that community programs are potential platforms for teaching and promotion of abstinence, thus the view that abstinence can be effectively supported and promoted in a variety of community platforms. The participants recommended development of specific community programs that intend to teach and promote sexual abstinence.

I think the community needs to stop telling people about sex but tell them about abstinence instead … (Male participant)

Yes, they need to call a teenager’s meeting and explain abstinence to them. They must not tell them about sex but about sexual abstinence instead, and keeping your virginity until you mature … (Female participant)

Community must call all youth and tell them about sexual abstinence. That if you choose to abstain, what will you get from it and what is good about it … (Male participant)

## Discussion

The study showed that the participants understood abstinence as ‘choosing not to have sex’, and the proportion of those who were abstinent was much higher than those who are sexually active (68% vs. 32%). The proportion of those that were dating was 46% vs. 54% that were not, and 22% of the participants were abstinent although dating.

### The value of abstinence

The value of abstinence seems to be appreciated by the participants as a primary tool to prevent consequences of sexual activity among young people. Factors that enable practice of abstinences include the intention to avoid STIs and unwanted pregnancies, a desire for a better future and religion, while the desire to experiment with sex, influence of social networks as well as abuse of alcohol and drugs were identified as barriers to abstinence. Although religion has been reported to influence abstinence as a positive sexual behavior, Odimegwu (2005) argues that religious commitment is more important than just affiliation, which means that there is a need to strengthen religious commitment among those who are religiously affiliated.

### Alcohol and sexual behavior

The influence of alcohol as a barrier to abstinence was similar to findings by other studies, which identified alcohol as a factor to risky sexual behaviors (Kabiru & Ezeh 2007; Kalichman, Simbayi, Kaufman, Cain & Jooste 2007; Simbayi, Kalichman, Jooste, Mathiti, Cain & Cherry 2004; Stueve & O’Donnell 2005). The finding that personal values and choices is a factor that enables the practice of abstinence is similar to the findings of Paradise, Cote, Minsky, Lourenco and Howland (2001) and Abbott and Dalla (2008). This implies that young people need to be encouraged toward an informed process of making their decisions, which is based on developing personal values.

### The role of the media

As with other findings, the sexual content of television was viewed as an enabler to sexual activities, which is similar to the findings of Collins *et al.* (2004) and Chandra, Martino, Collins, Elliott, Berry, Kanouse, *et al.* (2008). This results because television provides a considerable amount of information about sex without highlighting related risks and responsibilities. Being involved in a romantic relationship was identified as a barrier to sexual abstinence, and this was similar to the findings of Kabiru and Ezeh (2007), who concluded that a romantic relationship is a significant predictor of being involved in sexual activities, and that primary abstainers are least likely to be romantically involved.

Although some researchers promote the value of abstinence-only programs (Jemmott *et al.* 2010), others view this approach as not feasible as it deprives young people of other components of healthy sexuality (Santelli *et al.* 2006). Hindin and Fatusi (2009) argue that sex education should promote safe sexual behavior and not only messages that promote abstinence. This study did not seek to identify a preference for either.

### Sex education in the school curriculum

It was interesting that sex education at school was seen as both an enabler and a barrier to abstinence, but this may be explained by studies that identified limitations in teacher-delivered sex education in behavior change (Wight, Raab, Henderson, Abraham, Buston, Hart, *et al.* 2002), and that sex education did not produce the best outcomes in changing knowledge, perception and attitude of adolescent girls toward STIs (McManus & Dhar 2008). Further studies on the value of school-based sex education programs are recommended.

## Conclusion

The findings of the study showed that young people know what abstinence means, as well as its use and advantages, and that a variety of social factors contribute to decisions for and against abstinence. The advantages of abstinence, as published in the literature were well articulated. The role of the family, school and society in promoting sexual abstinence as a viable option in the prevention of STIs and teenage pregnancies was highlighted. All and these platforms can be used to promote safe sex practices that will contribute to positive sexual health outcomes for young people. Because the knowledge about abstinence, as well as its use was found to be good, the findings of this study suggest that this knowledge be elevated to application, that is, the learners to consider abstinence as an option in the prevention of STIs and teenage pregnancy.

## Recommendations

It is recommended that sexual abstinence be promoted as one of the viable options to prevent STIs and teenage pregnancies in this community. This can be achieved by developing and implementing abstinence-promoting programs in various platforms of this community.
